# Unusual coronary artery disease presentation: take the time to bury the hatchet and the stent, a case report

**DOI:** 10.1093/ehjcr/ytaf155

**Published:** 2025-04-15

**Authors:** Julien Rosencher, Cindy Marques, Ghilas Raouhal, Patrice Cacoub

**Affiliations:** Cardiology Department, Groupe Hospitalier Privé Ambroise Paré—Hartmann, 48 ter bld Victor Hugo, Neuilly-sur-Seine 92200, France; Sorbonne Université, Hôpital Pitié-Salpêtrière (AP-HP), 83 boulevard de l'Hopital, Paris 75013, France; Cardiology Department, Groupe Hospitalier Privé Ambroise Paré—Hartmann, 48 ter bld Victor Hugo, Neuilly-sur-Seine 92200, France; Sorbonne Université, Hôpital Pitié-Salpêtrière (AP-HP), 83 boulevard de l'Hopital, Paris 75013, France

**Keywords:** Case report, Cardiac CT angiography, Coronary artery disease, Coronary arteritis

## Abstract

**Background:**

Coronary artery disease (CAD) secondary to coronary arteritis (CA) is a rare and challenging condition to diagnose, often resulting in poor clinical outcomes. Conventional coronary angiography lacks the sensitivity to identify inflammatory causes, leading to underdiagnosis and inappropriate treatment. Advanced imaging techniques, particularly cardiac computed tomography angiography (CCTA), appear to be invaluable tools to correctly identifying CA as the underlying cause of atypical CAD.

**Case summary:**

We describe the case of a 74 year old patient without traditional risk factor who presented with chest pain, a positive clinical and electrical stress test. Given the highly atypical form of CAD on CCTA characterized by a diffuse, circumferential thickening of coronary arteries, an inflammatory cause was suspected. Large vessel vasculitis was confirmed by fluorodeoxyglucose-positron emission tomography scan (FDG-PET). Treatment with aspirin, statins, beta-blockers, and corticosteroids resulted in symptom resolution, with subsequent imaging showing regression of both vessels hypermetabolism and coronary arterial thickening, thus avoiding the need for coronary revascularization.

**Discussion:**

This case highlights the importance of multimodal imaging, particularly CCTA and FDG-PET, in diagnosing CA in patients with atypical CAD presentations. Early recognition and management of active inflammation can prevent unnecessary revascularization and improve clinical outcomes.

**Learning points**

Patient who presents with chest pain, abnormal cardiac stress test, and atypical presentation on coronary computed tomography angiography (CCTA).

- To understand the pivotal role of multimodal imaging in systemic vasculitis diagnosis and extent assessment.
- To be able to differentiate CCTA patterns of coronary arteritis from atherosclerotic coronary artery disease.
- To emphasize the importance of CCTA to guide coronary arteritis treatment.

## Introduction

Coronary artery disease (CAD) secondary to coronary arteritis (CA) is a rare condition, making diagnosis challenging and often leading to poor outcomes. While certain clinical features may suggest underlying vasculitis, conventional coronary angiography lacks specific diagnostic features, which frequently results in underdiagnosis and inadequate treatment. In this case, we present a patient with an atypical presentation of CAD, where coronary computed tomography angiography (CCTA) was essential in identifying an inflammatory cause.

## Summary figure

**Figure:**
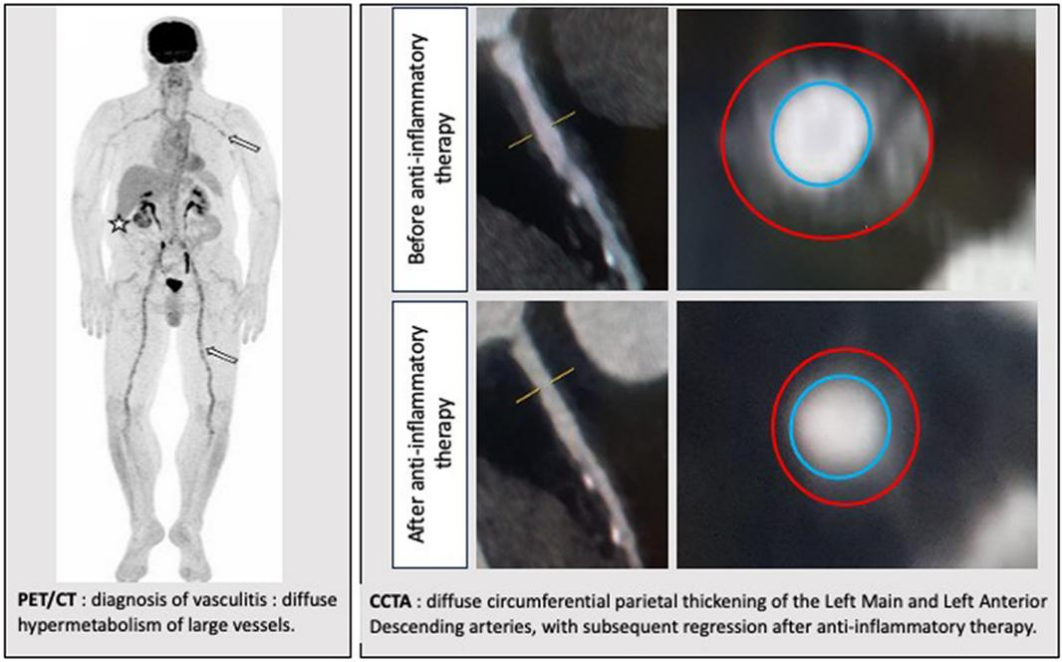
The arterial circumferential thickening is depicted as the area between the red (artery outer wall) and the blue (artery lumen) lines.

## Case presentation

A 74-year-old Caucasian patient was admitted for CCTA after experiencing Class II angina according to the Canadian Cardiovascular Society classification for 2 months. The electrocardiogram showed no T waves and ST-segment abnormalities, and the echocardiography results were normal. A positive exercise stress test revealed a down-sloping ST-segment depression concomitant with chest pain. He had no significant past medical history and no cardiovascular risk factors.

**Figure 1:**
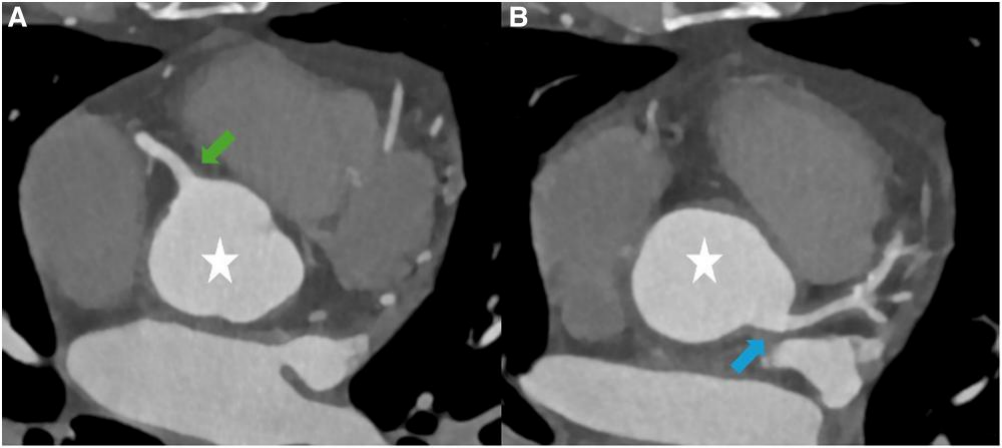
Computed tomography angiography of the ascending aorta and coronary ostia. Cross-sectional views (*A* and *B*) showing circumferential thickening of the ascending aorta (white star) extended to left main coronary artery and right coronary artery ostia (arrows) without significant stenosis.

**Figure 2:**
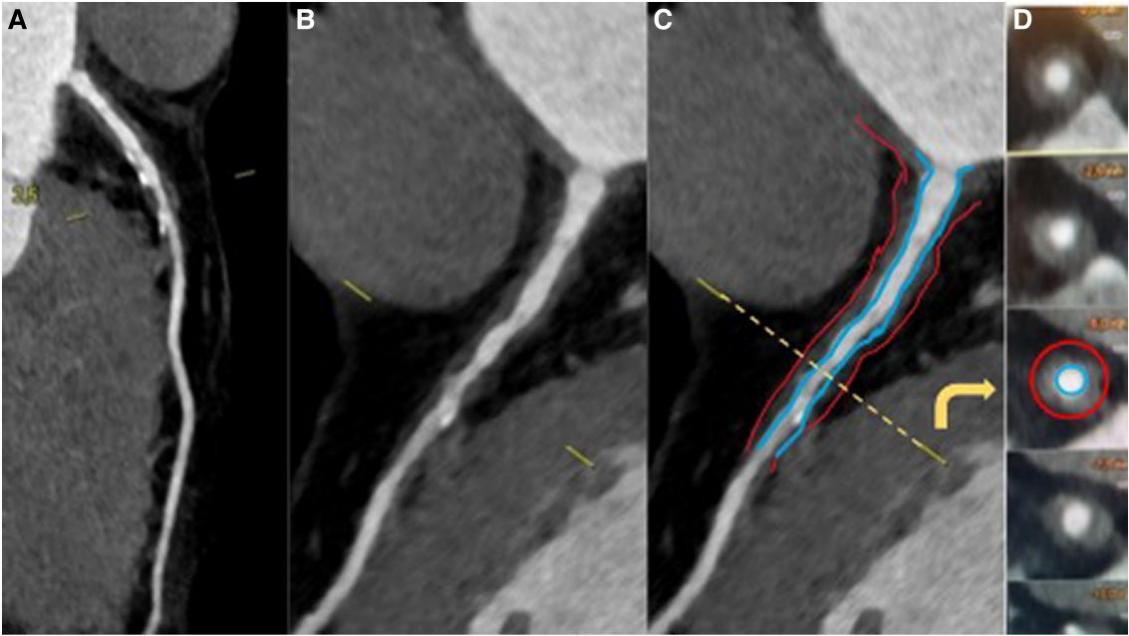
Coronary computed tomography angiography patterns of arteritis. Diffuse and circumferential thickening with low attenuation was observed from the left main ostium to the mid-left anterior descending artery. In the schematic representation, the circumferential thickening is depicted as the area between the red (artery outer wall) and the blue (artery lumen) lines. *(A–C)* Curved multiplanar reconstruction images obtained by cardiac computed tomography angiography. *(D)* Cross-sectional coronary image at the level of the yellow line.

Cardiac computed tomography angiography revealed a highly atypical form of CAD, characterized by a diffuse, circumferential thickening with low attenuation of the ascending aorta (*Figure 1*), the proximal left main coronary artery (LMCA), and Left Anterior Descending (LAD) artery (*Figure 2*). This arterial thickening resulted in a 50%–70% stenosis of the mid-LAD artery. Given this atypical presentation in a patient without traditional cardiovascular risk factors, an inflammatory cause was suspected. Therefore, we decided to postpone coronary revascularization and referred the patient to a specialized internal medicine unit for further evaluation.

## Diagnostic assessment

The clinical examination was unremarkable, with no changes in the patient’s general condition and no fever, sweating, headache, jaw claudication, or reduced visual acuity. The laboratory workup revealed no signs of biological inflammation, with neither an elevation in C-reactive protein levels nor an increase in erythrocyte sedimentation rate. A comprehensive immunological and infectious evaluation, including tests for antinuclear antibodies, antineutrophil cytoplasmic antibodies (ANCA), serum immunoglobulin G4 (IgG4), HIV, and syphilis serologies, were normal. Cardiac magnetic resonance imaging (CMR) showed no myocardial inflammation or late enhancement, with an estimated left ventricular ejection fraction of 65%.

**Figure 3:**
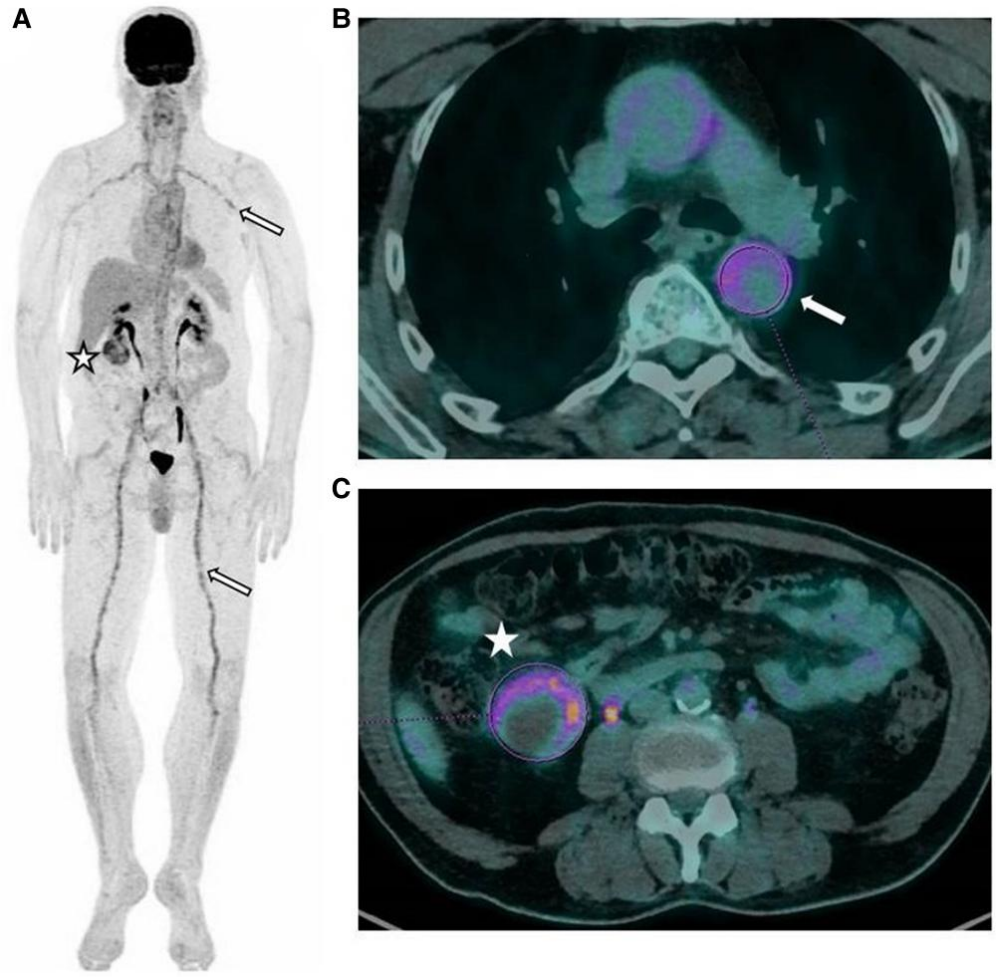
Large vessel arteritis diagnosis using positron emission tomography/computed tomography. Initial fluorodeoxyglucose-positron emission tomography scan maximum intensity projection (*A*) and fused positron emission tomography/computed tomography coronal views (*B*, *C*) revealed diffuse hypermetabolism of large vessels, consistent with vasculitis (arrows). Pathological fluorodeoxyglucose uptake was also observed in a heterogeneous right renal mass (star).

To investigate potential involvement of other organs, a fluorodeoxyglucose-positron emission tomography scan (FDG-PET) was performed, revealing signs of large vessel vasculitis with diffuse hypermetabolism of the aortic wall, as well as the supra-aortic, subclavian, coeliac, superior mesenteric, external iliac, femoral, and popliteal arteries suggestive of giant cell arteritis (GCA) (*Figure 3A* and *B*). The maximum standardized uptake value (SUVmax) ranged from 4.7 to 6.1. Fluorodeoxyglucose-positron emission tomography scan also detected a heterogeneous hypermetabolic mass in the right kidney, measuring ∼5.5 cm, with an SUVmax of 7.2 (*Figure 3C*). A temporal artery biopsy showed fibrous endarteritis with no evidence of GCA. Final diagnosis was classified as unlabelled arteritis, closely resembling GCA.

## Interventions

The patient was medically treated with aspirin, a high dose of statin, and a beta-blocker. The primary focus was to treat the right kidney mass, considering the hypothesis of paraneoplastic aortitis due to renal cancer. After a complete workup, no metastases were detected. Subsequently, the patient underwent a partial nephrectomy. Pathological examination confirmed a papillary carcinoma with clear margins. Follow-up FDG-PET scans at 2- and 5-months post-surgery showed no improvement in the pan-aortitis. Consequently, glucocorticoids were introduced at a dose of 0.7 mg/kg/day, followed by prednisone 10 mg/day for 1 month, then gradually reduced by 1 mg/month until reaching 5 mg/day.

## Follow-up

**Figure 4:**
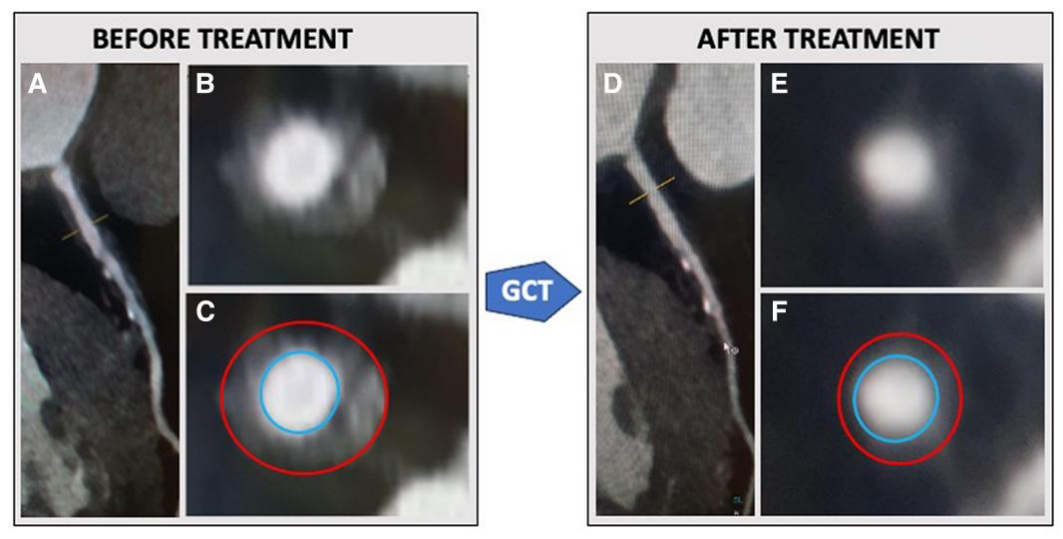
Regression of circumferential thickening of the left main and left anterior descending coronary arteries before (*A–C*) and after (*D–F*) glucocorticoids in cardiac computed tomography angiography. Unmarked (*B*, *E*) and marked (*C*, *F*) cross-sectional images of the left descending coronary artery corresponding to the yellow line level of the curved multiplanar reconstruction (*A*, *D*). In the marked images, the circumferential thickening is visualized between the red line (artery outerwall) and blue line (artery lumen). GCT, glucocorticoid.

The chest pain disappeared quickly with corticosteroid therapy and 4 months after the start of treatment, an FDG-PET scan showed regression of the aortic hypermetabolism (*Figure 4*). Furthermore, a 1-year follow-up CCTA revealed a notable regression of the coronary thickening in the LMCA and proximal part of the LAD artery with maximum stenosis under 50%.

## Discussion

Cardiac manifestations of systemic vasculitis are rarely seen in clinical practice, and coronary involvement is much less common than myocardial or pericardial involvement. Differentiating CA from atherosclerotic coronary disease is challenging, as the clinical presentation can be similar and manifests as typical angina, acute myocardial infarction, ventricular arrhythmias, or cardiac failure.^1^ Although uncommon, CA is associated with poor prognostic outcomes. In a large cohort of 1647 sudden cardiac death cases referred for pathological heart assessment, only 50 patients (3.0%) had non-atherosclerotic coronary pathology, with CA found in 6 (12%) of these cases.^2^

This case emphasizes the growing role of CCTA as a first-line tool in the diagnostic workup of CAD, allowing in our case for the identification of inflammatory patterns.^3^ The diagnosis of CA was suspected by analogy with aortitis, characterized by regular, circumferential wall thickening of more than 2 mm without calcified or non-calcified plaques.^4,5^ Additionally, a lack of traditional cardiovascular risk factors raised suspicion for an alternative aetiology, further supporting the diagnosis of CA. Despite the presence of symptomatic obstructive CAD, we decided not to perform coronary angiography and instead prioritized biological and non-invasive imaging techniques to better evaluate the potential underlying inflammatory process. Of note, although coronary angiography could have been the first-line investigation, it likely would not have provided a definitive diagnosis as it cannot visualize the circumferential thickening characteristic of inflammatory vascular diseases. In case of atypical presentation, the use of endovascular imaging such as intravascular ultrasound (IVUS) is now recommended to provide additional detail, including identification circumferential thickening with low attenuation.^4^

The role of multimodal non-invasive imaging in the diagnosis pathway of CA is increasingly recognized.^1^ In this case, the findings of diffuse coronary wall thickening on CCTA associated with artery wall hypermetabolism on FDG-PET were crucial in distinguishing inflammatory arteritis from atherosclerotic CAD. Although FDG-PET may not always differentiate vasculitis from atherosclerotic disease, it has become a pivotal diagnostic tool for systemic vasculitis, offering high positive and negative predictive values, and is also useful for evaluating treatment effects.^1,6^ Although not performed in this case, CMR can also detect inflammatory processes in both vascular walls and the myocardium.^1^ In similar scenarios, when faced with clinical atypia such as absence of vascular risk factors or unusual anatomical features like circumferential coronary involvement, aortic wall thickening, additional clinical, biological, and metabolic imaging investigations should be considered to avoid overlooking a non-atherosclerotic CA requiring timely and specific management.

The patient described in this case was a 74-year-old Caucasian male presenting with diffuse aortitis, notably involving the coronary arteries, without the evidence of stenoses or aneurysmal dilatation, and in a paraneoplastic context. Several causes of CA have been described, with the most common being Kawasaki disease, Takayasu arteritis, Polyarteritis Nodosa, and GCA.^1^ The description and imaging features of the several diseases that might present similarly are provided in *Table 1* for a comprehensive comparative framework. This table summarizes the key characteristics, imaging patterns, and distinguishing features commonly included in the differential diagnosis of CA. In the present case, several factors, including the patient’s gender, age, ethnicity, and the recent onset of aortitis without structural changes, argue against a diagnosis of Takayasu arteritis, which typically affects younger females with typical ostial coronary artery involvement.^7,8^ Similar cases of IgG4-related disease have been described, with diagnosis established by CCTA in most of the cases.^4^ However, IgG4-related disease was also ruled out based on normal serum IgG4 levels and the absence of other clinical manifestations suggestive of this condition, such as lymphadenopathy, or pancreatic, pulmonary, orbitary and neurological involvement. A diagnosis of GCA could not be confirmed as the patient did not present cranio-cephalic symptoms (headaches, scalp tenderness, and jaw claudication), and the temporal artery biopsy showed no histological evidence of GCA. Consequently, the diagnosis was classified as unlabelled arteritis, closely resembling GCA, given the absence of resolution after cancer treatment and the excellent response to corticosteroid therapy.

**Table 1:** Differential diagnosis of coronary arteritis

Disease	Description	Common imaging features	Differentiating features
Atherosclerotic CAD	Progressive narrowing of coronary arteries due to lipid and plaque deposition	Eccentric stenosis, calcifications, and plaque formation on angiography or CCTA	Presence of traditional risk factors (diabetes and hypertension), absence of systemic inflammation
Takayasu arteritis	Large vessel vasculitis affecting the aorta and its branches, often seen in young females	Long, smooth stenosis or occlusion, arterial wall thickening (>2 mm) on CT or MRI, circumferential wall thickening with FDG-PET hypermetabolism	Often involves aortic arch vessels; systemic symptoms such as fever, malaise, and claudication; functional and morphological sequalae of previous flares
Giant cell arteritis (GCA)	Large vessel vasculitis commonly affecting older individuals	Circumferential wall thickening with FDG-PET hypermetabolism in the aorta and major branches	Associated with temporal artery tenderness, elevated C-reactive protein; possible association with polymyalgia rheumatica; histological diagnosis on temporal artery biopsy
IgG4-related disease	Multi-systemic fibro-inflammatory disease	Diffuse arterial wall thickening, delayed enhancement on contrast-enhanced imaging	Elevated IgG4 levels, specific lymph node or visceral involvement; characteristic histopathology on biopsy
Ankylosing spondylitis	Chronic inflammatory disease affecting the axial skeleton	Aortic root and ascending aorta thickening, valvular involvement on CT/MRI	Predominant musculoskeletal symptoms; sacroiliitis on imaging, HLA-B27 positivity
Atrophic polychondritis	Rare systemic autoimmune disease affecting the cartilages of the ears, nose, and respiratory tree	Aortic insufficiencies complicating dilatation of the aortic root, aneurysmal developments in the ascending thoracic aorta	Predominant cartilage involvement
Behçet's disease	Multi-systemic inflammatory disorder causing vasculitis of all vessel sizes	Aneurysms, occlusions, and stenoses of large vessels on angiography	Oral/genital ulcers, uveitis, positive pathergy test, HLA B51 positivity
ANCA-associated vasculitis	Small vessel vasculitis associated with anti-neutrophil cytoplasmic antibodies (ANCA)	Inflammation of small vessels, sometimes with microaneurysms, rarely affecting large vessels	Positive ANCA (p-ANCA or c-ANCA), common renal, ear nose and throat, pulmonary, or peripheral nerve involvement
Cogan’s syndrome	Rare autoimmune disease involving eyes and inner ears, with possible large vessel vasculitis	Aortic wall thickening and aneurysms, FDG-PET hypermetabolism	Associated with corneal and cochleovestibular damage
Erdheim-Chester disease	Rare non-Langerhans histiocytosis affecting multiple systems, including large vessels	Peri-aortic ‘coated aorta’ sign, FDG uptake in soft tissue surrounding vessels	Bilateral sclerotic bone lesions, CD68-positive histiocytes, BRAF mutation in many cases
Rheumatoid arthritis (RA)	Chronic inflammatory disease primarily affecting joints	Aortic wall thickening, coronary vasculitis on imaging in advanced disease	Predominant joint involvement, positive rheumatoid factor (RF), and anti-cyclic citrullinated peptides
Sarcoidosis	Granulomatous inflammatory disease affecting multiple organs	FDG-PET shows patchy vascular hypermetabolism, lymphadenopathy	Pulmonary or visceral involvement, elevation of angiotensin converting enzyme, non-caseating granulomas on biopsy
Polyarteritis Nodosa (PAN)	Necrotizing vasculitis of medium-sized arteries	Microaneurysms and segmental stenoses of ascending thoracic aorta on CT or MR angiography	Systemic symptoms (e.g. abdominal pain and neuropathy), association with hepatitis B
Kawasaki disease	Vasculitis seen in children, involving coronary arteries	Coronary aneurysms or ectasia, occasionally with thrombosis	Predominantly occurs in children; fever, rash, and conjunctivitis
Systemic lupus erythematosus (SLE)	Chronic autoimmune disease affecting multiple organs	Vasculitis-related stenoses or thrombosis on angiography	Systemic features (e.g. rash and joints). positive ANA, anti-dsDNA, anti-Sm, and complement consumption
Infectious arteritis	Arteritis caused by bacterial or fungal infections	Focal arterial wall thickening, possible abscess formation, or aneurysms	Positive blood cultures or serology, response to antibiotics/antifungal therapy
Tuberculosis	Chronic mycobacterial infectious that can cause aortitis in its advanced stages	Aortic wall thickening, pseudoaneurysms, and calcifications	Pulmonary or lymph node involvement, history of contagion, positive tuberculin skin test/interferon gamma release assay, granulomas with caseation on biopsy
Syphilis	Chronic infection, potentially causing tertiary syphilitic arteritis	Irregular vessel wall thickening with aneurysmal dilation on imaging	Positive serologic veneral disease research laboratory/treponema pallidum hemagglutination assay (VDRL/TPHA), history of untreated syphilis

Management of CA is complex and depends on its aetiology. In our patient, the accurate diagnosis prevented unnecessary revascularization procedures, which could have been associated with poor prognosis, higher risks of restenosis, or graft failure in an inflamed vascular bed.^8,9^ Early imaging guided diagnosis and the control of active inflammation seem to be crucial for improving clinical outcomes. Indeed, treatment with glucocorticoids led to significant improvement in both metabolic and coronary stenosis. At 12 months of follow-up, the patient no longer presented with chest pain or dyspnoea. Revascularization was not performed, as the patient showed regression of coronary thickening and inflammation on imaging, with no clinical manifestations of ischaemia. Moreover, if the diagnosis had not been established using both imaging modalities, the renal cancer could have progressed, preventing an upfront curative surgical intervention, and the aortitis could have advanced, leading to structural changes (aneurysmal dilations and stenosis) with functional sequelae.

## Conclusion

The diagnosis and management of CA remain challenging, particularly in differentiating it from atherosclerotic CAD. An atypical presentation of symptomatic obstructive CAD, such as diffuse circumferential coronary thickening and low-attenuation patterns on CCTA, should prompt careful evaluation before proceeding with coronary angiography or revascularization. Integrating PET-FDG findings of active inflammation with clinical and laboratory investigations enables timely diagnosis and targeted treatment. Early recognition and inflammation control are crucial for optimizing clinical outcomes and avoiding unnecessary or potentially harmful revascularization procedures.

## Lead author biography


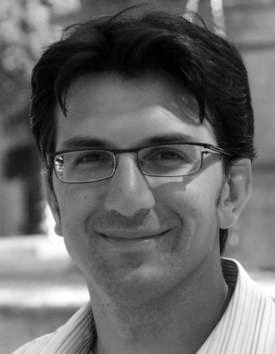


Julien Rosencher is working in the catheterization laboratories and in the cardiovascular imaging laboratories at the Groupe Hospitalier Privé Ambroise Paré—Hartmann, France. Dr Rosencher has a strong interest in clinical education with mentorship in both laboratories. He is also involved in the French Liberal Cardiologist Association (CNCF).

## Data Availability

All data exposed in this case report were acquired from our institution, after obtaining informed consent from the patient.

## Notes

**Consent:** The patient consented to the publication of this case report in compliance with the COPE guidelines.

**Funding:** None declared.
